# Effect of Different Cooking Methods on Lipid Content and Fatty Acid Profiles of *Mytilus galloprovincialis*

**DOI:** 10.3390/foods10020416

**Published:** 2021-02-13

**Authors:** Francesca Biandolino, Isabella Parlapiano, Giuseppe Denti, Veronica Di Nardo, Ermelinda Prato

**Affiliations:** 1 National Research Council, Water Research Institute (CNR-IRSA), Via Roma, 3, 74123 Taranto, Italy; isabella.parlapiano@irsa.cnr.it (I.P.); giuseppe.denti@irsa.cnr.it (G.D.); linda.prato@irsa.cnr.it (E.P.); 2 Department of Biomolecular Sciences, University of Urbino “Carlo Bo” Via Donato Bramante, 28, 61029 Urbino, Italy; veronikadinardo@gmail.com

**Keywords:** mussels, cooking, lipids, fatty acids, nutritional quality indices, Mediterranean Sea

## Abstract

The effect of cooking (barbecue-grilling, boiling, microwaving, oven cooking and frying) on lipids, fatty acids (FAs) and lipid quality indices of the mussel *Mytilus galloprovincialis* was investigated. In general, all processing methods significantly (*p* < 0.05) modified the fatty acid profiles of mussels, although with major changes in fried samples, which exhibited the lowest saturated fatty acids and *n*-3 and highest polyunsaturated (PUFA) and *n*-6 FAs content. A significant decrease in the *n*-3 PUFA from the raw sample to five cooking methods was observed. The *n*-3/*n*-6 ratio decreased from raw (6.01) to cooked mussels, exhibiting the lowest value in fried ones (0.15). C20:5 *n*-3 and C22:6 *n*-3 significantly decreased during all cooking processes, and overall in fried mussels. It can be concluded that cooking does not compromise the nutritional quality of mussels except with frying, although it resulted in a decrease of the atherogenic and thrombogenic indices.

## 1. Introduction

Seafood represents one of the healthiest foods due to its nutritional benefits, and it has been recommended in several dietary regimes. It is widely consumed in many parts of the world for its high quality protein content, low caloric value, and low saturated fat and carbohydrate. More than one billion of the poorest people rely on fish and seafood as a primary protein source [1]. Its important potential in human nutrition is mainly due to the significant amounts of *n*-3 series polyunsaturated fatty acid (PUFA), especially eicosapentaenoic acid (EPA) C20:5 *n*-3 and docosahexaenoic acid (DHA) C22:6 *n*-3, with well-established beneficial properties for human health. They are likely to lower the risk of heart diseases in adults; to prevent various diseases such as blood pressure, coronary heart disease, cancer, type 2 diabetes and inflammatory disease; and are important for neurodevelopment in infants and young people [2,3].

Humans do not have specific enzymes to introduce double bonds at the *n*-3 or *n*-6 positions, necessary to obtain precursors for the synthesis of more highly unsaturated *n*-3 and *n*-6 fatty acids (FAs) [4]. So, these FAs are considered ‘‘essential fatty acids’’ (EFAs) that need to be obtained through food.

The primary source of EPA and DHA is seafood and products derived from it, even though amounts can vary significantly [5,6,7,8,9,10,11].

Among them, bivalve mollusk, a seafood very much appreciated by consumers, provides a great amount of *n*-3 fatty acids and low amount of saturated fatty acids (SFA) [5,9,12]. Moreover, marine bivalves also constitute a sustainable type of food production since they feed mainly on marine phytoplankton that occurs in the ecosystem, so no additives such as vitamins and antibiotics are added.

The global production of marine bivalves for human consumption has amounted to more than 15 million tons per year (average period 2010–2015). Most of the marine bivalve production (89%) comes from aquaculture and only 11% comes from wild fisheries. Among the EU member states, Italy is one of the largest edible bivalve producers, after Spain and France [13]. Within bivalve mollusks, mussels are the most successful species in Mediterranean countries, for their taste and their competitive price compared to other bivalves and fish [14].

Bivalve species are preferably consumed live/raw or lightly cooked, increasing the risk for human health as bivalves can concentrate pathogenic micro-organisms such as bacteria, human viruses, toxins from harmful algal blooms and chemical contaminants from the water column (since they have filter feeding activity) [15].

*Mytilus galloprovincialis* is a representative mussel species of the Mediterranean Sea, and in Italy in 2015, 63,700 tons were produced, after China and Spain [16].

In Italy, *M. galloprovincialis* mussels are eaten raw with guaranteed freshness and sanitary parameters by authorized points of sale that follow European Parliament and Council (*EC*) Regulation no. 853/2004, and these mussels are also largely appreciated when processed by different cooking methods before consumption. The cooking process (boiling, grilling, baking and frying) can be advantageous in many ways, and can positively affect the food color, taste and flavor, making them more attractive to consumers and destroying bacteria or other harmful micro-organisms [17].

On the other hand, the cooking process is responsible for several alterations to nutrient structure, leading to a reduction in the nutritional value of the final product [18]. Some of the principal changes that occur during processing and final preparation of cooked food are due to oxidation phenomena [19], for which PUFA are more susceptible during heating than SFA. Most of the literature data on this topic refers to different fish species [20,21,22], while only a few studies exist on bivalves and in particular on *M. galloprovincialis* cooked using several common domestic practices such as boiling, roasting and frying [23,24,25]. Studies on nutritional data for *M. galloprovincialis* are mostly focused on raw samples [5,7,9,10] and not on the cooked ones.

Consumer trends towards healthier products are becoming more popular, as they are becoming aware about their nutritional needs, and so they want to know which cooking methods are suitable to preserve food nutrients.

The present study aimed to investigate the effects of five cooking methods (barbecue-grilling, boiling, microwaving, oven cooking, frying) on both the lipid content and fatty acids profile of the *M. galloprovincialis* mussel, from the Ionian Sea (Mediterranean Sea). The values obtained in the cooked samples were compared with the values found in raw bivalve.

This species was chosen for this exploration because there are few reports on the changes that occur due to culinary preparation. Thus, this investigation can provide great insights into the choice of culinary preparation method, depending on the fat content of this bivalve. The results could help consumers to choose the healthiest cooking methods for mollusks.

## 2. Materials and Methods

### 2.1. Sample Origin, Preparation and Processing

A total of 200 live mussels (*M. galloprovincialis*), with a length of 4–6 cm and weight of 5–16 g, were collected at a mussel plant in the Ionian Sea (central Mediterranean, southern Italy: 40°25′54″ N, 17°1′22″ E) in July 2019. Samples were immediately transported in a plastic container with ice to the laboratory. Upon arrival (within 1 h), the mussels were washed with tap water several times to remove epibionts.

Mussels were randomly divided into six groups and each group consisted of about 45 individuals, with 15 samples for each replicate (*N* = 3).

The mussels in the first group were uncooked, while the other five groups were cooked in the following methods: barbecue-grilling, boiling, microwaving, oven cooking and frying. Soft tissues were cleaned and washed with distilled water to remove extraneous material before cooking.

Barbecue-grilling was performed by cooking the mussels over wood on an open grill and slow cooking over a fire for a 5–10 min, at a temperature of 80 °C.

Boiling was performed in a small amount of water at 85–90 °C (water temperature) for about 10 min until the whole-in-shell mussels were removed from the pot at the exact time of opening of the valves.

Microwaving was done by placing the whole-in-shell mussels in the microwave and cooking at regular power (750 W) for 3 min; the mussels were then turned over and the cooking process continued for another 1 min. Mean center temperature after cooking was 92.5 ± 1 °C.

Oven cooking was performed in a conventional oven preheated to 180 °C. Once this temperature was reached, mussels were positioned on a rack in the bottom third of the oven. The mussels were cooked for about 10 min, until the shells opened.

Frying of mussels was performed in a domestic frying pan with 1 L capacity. Commercial sunflower oil 0.3 L was added into the frying pan and heated until it reached an approximate temperature of 170 °C. In order to achieve uniform cooking, mussels were fried for 4 min with occasional turning. After cooking, a dry absorbent no. 1 Whatman filter paper was placed under the cooked samples to absorb excessive oil. Sunflower oil was purchased from a local market. The fatty acids profile of the sunflower oil was analyzed before and after cooking.

All processing methods were carried out without the addition of any ingredients. After cooking, all samples were placed for 3 min on absorbent paper towels.

The fresh and cooked mussels were minced, homogenized with an Ultra-Turrax IKA Werke, mixed to obtain uniform initial material, vacuum-packed, and deep-frozen for 3 days. Then, the samples were freeze-dried, ground and stored at −20 °C until analysis, which occurred within two weeks at the latest.

### 2.2. Moisture, Lipid and Fatty Acid Analyses

Moisture percentage was determined by drying the sample in an oven at 105 °C overnight until a constant weight was obtained. About five grams of sample was placed in an aluminum dish and preweighed (W1); then it was removed from the oven, cooled down in a desiccator and reweighed (W2).

Equation (1) was applied to calculate the moisture percentage:

Moisture (%) = (W1 − W2)/W1 × 100(1)

Total lipid was extracted from both the raw and cooked mussels via chloroform:methanol (2:1, *v/v*) solvent system [26]. The lipid content was gravimetrically determined.

The fatty acids (FAs) of mussels were determined as fatty acid methyl esters (FAME) after extraction using the direct transmethylation method with 14% boron trifluoride (*v/v*) in methanol. FAMEs were analyzed by gas chromatography using an HP 6890 series GC (Hewlett Packard, Wilmington, DE, USA), equipped with a flame ionization detector (GC-FID). FAMEs were separated with an Agilent HP-88 column (60 m × 0.25 mm id, 0.2 µm). Helium (purity 99.99%) was used as the carrier gas at a flow rate of 1 mL/min. The column temperature program was as follows: 150 to 250 °C at 4 °C/min and then held at 250 °C.

Fatty acid methyl esters were identified by comparing their retention times with those of a mixture of fatty acid methyl ester standards (Supelco 37 Component FAME Mix; Supelco Inc., Bellefonte, PA, USA). The results were expressed as the percentages of peak areas.

FAMEs were quantitated in g/100 g tissue by internal standard quantification, employing methyl nonadecanoate (98% purity, Sigma-Aldrich Chemicals, Saint Louis, MO, USA). The results were expressed as mg/100 g dry weight.

### 2.3. Lipid Nutritional Quality Indices (LNQI)

The nutritional quality of the lipid fraction was evaluated by three indices from the composition data in fatty acids.

The atherogenic (AI) and thrombogenic (TI) indices are important tools, from a consumer point of view, to estimate the probability of developing coronary heart diseases, and are calculated according to Equations (2) and (3) [27], where MUFA is monounsaturated fatty acids:

AI = (12:0 + (4 × 14:0) + 16:0)/(∑MUFA + ∑PUFA (*n*-6) + (*n*-3)) (2)

TI = (14:0 + 16:0 + 18:0)/((0.5 × ∑MUFA) + (0.5 × ∑PUFA (*n*-6)) + (3 × ∑PUFA (*n*-3)) + (*n*-3)/(*n*-6)) (3)

The hypocholesterolemic/hypercholesterolemic index (H/H) of both raw and cooked mussels was evaluated according to Equation (4) [28]:

H/H = (18:1 *n*-9 + 18:2 *n*-6 + 20:4 *n*-6 + 18:3 *n*-3 + 20:5 *n*-3 + 22:5 *n*-3 + 22:6 *n*-3)/(14:0 + 16:0) (4)

### 2.4. Statistical Analysis

Results are expressed as the mean of triplicate trials ± standard deviation. Data were analyzed for normality and variance homogeneity through the Kolmogorov–Smirnov and Levene’s tests, respectively. A one-way analysis of variance (ANOVA) was performed to determine the difference in nutrients between raw and cooked samples. A post-hoc Tukey test was performed to find significant differences between means. Differences were considered significant when *p* < 0.05.

## 3. Results and Discussion

### 3.1. Moisture and Lipid Content

**Figure 1:**
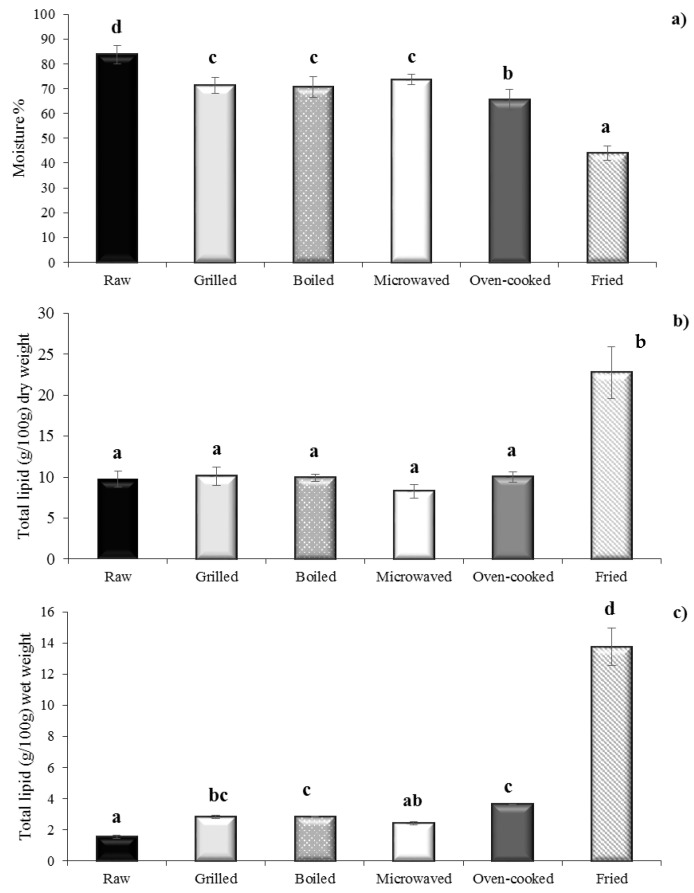
(**a**) Moisture percent (%), (**b**) lipid content (mg/100 g dry weight basis) and (**c**) lipid content (mg/100 g wet weight basis) of raw and cooked mussels (*Mytilus galloprovincialis*). Different letters over bars indicate significant differences among means (*p* < 0.05).

Changes in moisture (%) and lipid content (g/100 g dry weight and g/100 g wet weight) of the mussel samples in relation to the five culinary preparations are reported in Figure 1. The raw (fresh) mussels exhibited moisture and lipid content in line with those reported by several authors, characterized by high moisture and low lipid content [5,9,10,23]. All the cooking methods resulted in a significant decrease in moisture with the following order: frying > oven cooking > boiling = grilling = microwaving. The decrease in moisture content, caused by water evaporation [20], depended on the temperature achieved. Similar to our results, Weber [20], in a study on the effect of seven cooking methods on the proximate and fatty acids composition of silver catfish (*Rhamdia quelen*) found that the frying process caused the highest moisture loss.

This phenomenon is reported as the main change that determines a significant increase in protein, fat and ash concentration in cooked samples [29]. Accordingly, in this study, the lipid content of mussel significantly increased in all cooking methods when compared with the raw sample (*p* < 0.05) (Figure 1), except for microwaving, showing values, expressed on a wet weight basis (product ready to eat), in the range of 2.40 g/100 g wet weight (ww) (microwaved) −13.75 g/100 g ww (fried).

Such alterations were more evident for fried mussels, which exhibited the highest moisture loss and the highest lipid amount compared with raw and other samples (*p* < 0.05). In particular, the lipid content of fried mussels, with a value of 13.75 ± 1.2 g/100 g ww, was up to more than eight times the lipid content of the raw mussel (1.55 ± 0.10 g/100 g ww) (Figure 1). This result agrees with other authors who stated that the immersion in oil represents a crucial culinary method to affect the fat level of meat, as a result of absorption of frying oil (in this case, sunflower) by the mussel tissues and partial loss of water by evaporation [20,22,30].

Kalogeropoulos [23], in a study on the effect of domestic pan frying on *M. galloprovincialis*, found a considerable decrease in moisture content from 82.2% in raw sample to 57% in fried, and an increase of total fat from 22.7 g/kg ww to 110.2 g/kg ww in raw and fried, respectively. Felici [31], in a study on the effect of cooking on quality traits of cooked oysters (*Crassostrea gigas*), also observed lower moisture content and higher fat content than the raw ones. Bejaoui [32], in a study on the effect of culinary treatments on the fatty acid composition of a commercial clam (*Ruditapes decussatus*), reported similar changes in moisture and lipid content of clams after grilling and frying. A number of studies on the effect of cooking on bivalves, crustaceans and fish reported similar results [24,25,29,33,34,35]

Therefore, based on the results from this study, microwaving appears to be the healthiest cooking method, since the fat content is preserved.

### 3.2. Fatty Acids Profile

In order to verify the effect of the cooking process on the fatty acids content, the results were calculated on the dry basis, thus eliminating the influence of moisture (Table 1). Moreover, to provide important information to consumers, the most important FAs are also expressed on a wet weight basis (product ready to eat) (Table 2). So, the minimal changes observed passing from wet weight to dry weight must be a consequence of the water loss produced by these cooking processes. In addition, since estimations of mussel nutritive value based on mass units are comparatively scarce, and in order to enable a comparison of the results from this study with international and national research, the data were also expressed as a percentage of fatty acids per total FAs (Table 3).

**Figure 2:**
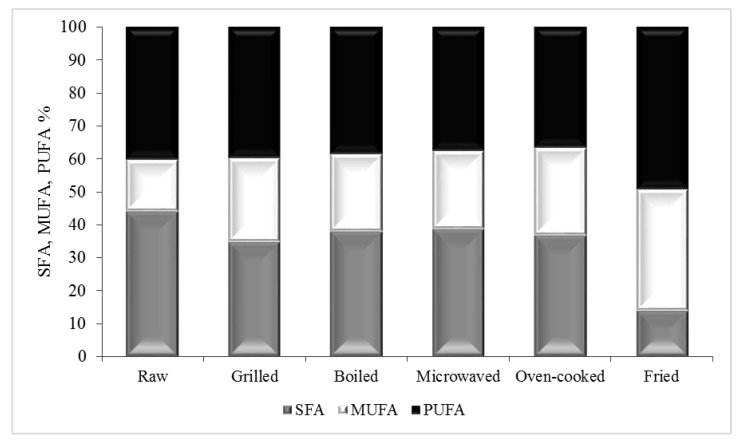
Proportion of saturated (SFA), monounsaturated (MUFA) and polyunsaturated fatty acids (PUFA) in raw and cooked mussels.

Twenty-three fatty acids exceeding a minimum of 0.1% total FAs in a minimum of one sample were identified (Table 1, Table 2 and Table 3). Despite the low lipid content of mussels, they constitute an important source of fatty acids. The raw mussels exhibited a favorable fatty acid profile, with a dominance of saturated fatty acids (SFA, 43.9%) and PUFA (40.2%), followed by lower amounts of MUFA (15.9%) (Table 3, Figure 2). These data are in agreement with those obtained by Biandolino [5] and Prato [9] on the same and other bivalve species.

Dietary fats are important nutrients for many vital functions; they are a source of energy for many processes in the body, support cell growth, etc. However, during cooking, nutrients are likely to undergo changes in their structure and content, affecting the nutritional quality of the final product [36]. After cooking, SFA, MUFA and PUFA changed their proportion of major fatty acids significantly (*p* < 0.05).

**Figure 3:**
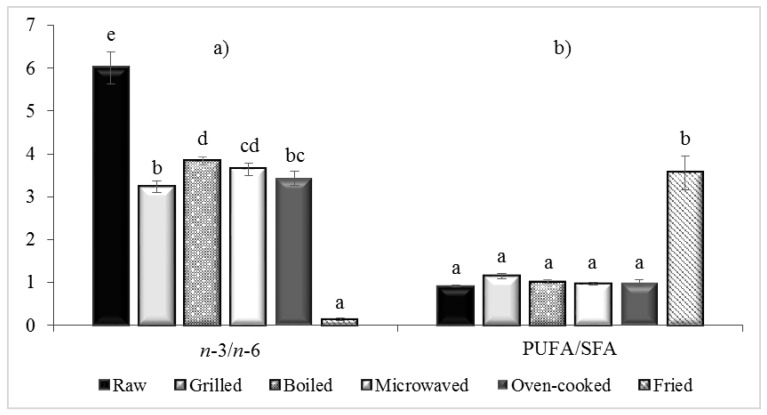
(**a**) *n*-3/*n*-6 ratio and (**b**) PUFA/SFA ratio of *Mytilus galloprovincialis,* raw and cooked. PUFA, polyunsaturated fatty acids; SFA, saturated fatty acids. Means with different letters on the bars differ significantly (*p* < 0.05).

All cooking methods determined a significant decrease of SFA within the range of 13.9–38.6% of total FAs. The lowest proportion of SFA was found in the fried sample, about 30% lower with respect to raw sample (Table 1, Table 2 and Table 3, Figure 2). The MUFA proportion increased significantly after all cooking methods (*p* < 0.05), showing the highest content in fried sample, accounting for 36.77% of total FAs (3538.4 mg/100 g ww) by over two-fold higher compared to the raw mussel value (15.9%) (Table 2, Figure 3).

Though significant amounts of MUFAs and SFAs were found, PUFAs were the main fraction in the most cooking treatments (Table 1, Table 2 and Table 3, Figure 2). Grilling, boiling and microwaving did not affect the proportion (%) of PUFA; conversely, fried mussel showed a significant increase in PUFA proportion (49.3%) and oven-cooked mussel a decrease (37.6%), compared with the raw sample (40.18%) (*p* < 0.05) (Table 3, Figure 2). On a dry weight basis, most of the cooked samples presented a similar PUFA content to that of raw mussels (2747.80 mg/100 dry weight (dw)). Microwave cooking resulted in the lowest value of PUFAs with 2172.45 mg/100 g dw (Table 1), while on a wet weight basis (ready to eat), the raw mussels (Table 2) showed the lowest content (*p* < 0.05).

The significant decrease of SFA and increase of MUFA from the raw sample to the cooked ones (overall fried samples) were shown by Kalogeropoulos [23] for mussels fried in virgin olive oil, and Felici [31] for cooked oyster (in olive oil and gratin), and in several other studies on fish [20,35,37].

On the other hand, Bejaoui [32] reported for the clam *Ruditapes decussatus*, after frying with corn oil, not only was there an increase of MUFA, but also an increase of SFA. Ghribi [12] observed in the edible shellfish Noah’s Ark (*Arca noae*) a constant proportion of SFA after grilling and a slight increase after boiling and frying (in extra virgin olive oil). MUFA showed a significant increase only in fried *Arca noae*, while the PUFAs proportion remained stable after steaming, boiling, grilling and frying.

SFA intake, especially palmitic acid (C16:0) and myristic acid (C14:0), is associated with cardiovascular disease (CVD), contributing to the increase of low-density lipoprotein (LDL) cholesterol.

Since 1970, a reduction in the intake of SFA has been recommended for reducing the risk for CVD [38]. However, the relationship between SFA consumption (types and amounts) and risk of CVD in adults is still unclear and controversial. It is also associated with development of cancer, diabetes mellitus, infections and chronic liver disease mortality [39].

The increase in MUFAs should not be regarded as a negative fact; indeed, in the last years, MUFAs have received considerable attention because they are recognized as being beneficial for health. Several studies reported a clear association between a Mediterranean diet rich in MUFA from olive oil and cardiovascular heart disease risk reduction [40]. The replacing of SFAs with MUFAs has been shown to have beneficial effects on blood cholesterol and other health-related outcomes and, in particular, a reduction of cardiovascular risk factors (total cholesterol, LDL cholesterol and triglycerides) and increases in HDL (High-Density Lipoprotein) cholesterol [38] have been observed.

The most abundant fatty acids found in raw mussels were palmitic acid (C16:0, 28.2%), EPA (C20:5 *n*-3, 12.9% of total FAs), DHA (C22:6 *n*-3, 11.1% of total FAs), palmitoleic acid (C16:1, 8.5% of total FAs) and myristic acid (C14:0, 8.25% of total FAs). These findings are in agreement with those obtained by Kalogeropoulos [23], Biandolino [5] and Prato [9], for *M. galloprovincialis*.

All cooking methods affected the fatty acid profile of mussels (*p* < 0.05). It is not surprising that the cooking process that most affected the fatty acids profile of mussels was pan frying, which showed major changes when compared to raw samples. This can mainly be attributed to the absorption of oil during frying, with the changes reflecting the fatty acid composition of the frying oil and oxidation of the mussel fatty acids [35,41]. For these reasons, this cooking process will be discussed separately.

For all remaining cooking methods, when compared to raw samples, the most significant increases were observed for palmitoleic acid (C16:1), eicosenoic acid (C20:1 *n* 9), linolenic acid (C18:3 *n*-3) and arachidonic acid (ARA, C20:4 *n*-6). The significant decreases were found in myristic acid (C14:0), palmitic (C16:0), stearidonic acid (C18:4 *n*-3), EPA, docosapentaenoic acid (DPA, C22:5 *n*-3) and DHA. Oleic acid remained stable after the other cooking processes. Similar results are reported by Bejaoui [32] for cooked clam *Ruditapes decussatus*, Felici [31] for oyster *Crassostrea gigas*, and by Otles and Sengor [24] for mussel *M. galloprovincialis*.

The frying determined a totally different fatty acids profile compared with the raw sample and all remaining cooking methods (*p* < 0.05). It was characterized by a strong decrease of myristic acid (C14:0, from 8.2% in the raw to 1.4% of total FAs, in the fried) and palmitic acid (C16:0, from 28.2% to 10.9% of total FAs) that justify the lower proportion of SFA. A significant decrease was observed also for palmitoleic (C16:1, from 8.46% to 3.13% of total FAs), ARA (C20:4 *n*-6, from 3.2% to 1.0% of total FAs), EPA (C20:5 *n*-3, from 12.9% to 2.4%), DPA (C22:5 *n*-3, from 1.47% to 0.17% of total FAs) and DHA (C22:6 *n*-3, from 11.1% to 2.16% of total FAs). Oleic acid (C18:1 *n*-9) greatly characterized the fried product, increasing by about 15 times the value of raw sample, from 4.50% (48.78 mg/100g ww) to 29% of total FAs (2779.31 mg/100 g ww) in raw and fried, respectively, contributing to the highest MUFA content (Table 2 and Table 3). The major change observed after frying was the significant increase of linoleic acid (LA, C18:2 *n*-6) from 1.77% (19.15 mg/100 g ww) in raw sample to 41.57% of total FAs (3999.68 mg/100 g ww) in fried mussel (Table 1 and Table 2), which determined the increase of PUFA. Obviously, the change of the fatty acids composition depends on the oil used for frying. The results confirm that the fatty acids of cooking oil, in this case sunflower oil, rapidly penetrate the flesh even within 3 min of cooking [42], affecting the fatty acids composition of the mussel. The analysis of fatty acids of the sunflower oil (Table 2) before and after frying evidenced this fact. Before frying, sunflower oil is characterized by richness in linoleic acid (59.16 mg/100 ww) followed by the oleic acid (30.21 mg/100 ww). After frying, these fatty acids significantly decreased (*p* < 0.05), migrating in large amount to the fried mussel. Therefore, frying in sunflower oil markedly increased the oleic and linoleic acid contents of the mussels, and simultaneously determined the decrease of the remaining fatty acids. An exchange of fatty acids was established between the frying oil and the animal’s tissue, as can also be seen from the fatty acid profile of the sunflower oil before and after frying (Table 2).

These results are in agreement with those reported by several authors, which reported a significant increase in C18:1 *n*-9 and C18:2 *n*-6 after frying, e.g., Czech [25], for *Mytilus edulis,* found values of oleic acid and linoleic acid of 20.82% and 58.56% of total FAs. Bilgin [43], for rainbow trout (*Oncorhynchus mykiss*) fried in sunflower oil, reported the highest values of oleic and linoleic acid, and likewise, Candela [44], for three types of fish (sardine, mackerel and salmon).

The raw mussel *M. galloprovincialis* is a good source of *n*-3 PUFA with low content of *n*-6 PUFA, as reported in previous studies [5,9,23,24]. The data of this study showed that the level of *n*-3 PUFA was always higher than the level of *n*-6 for all types of cooking, except for fried sample (Table 1, Table 2 and Table 3). However, the main effect of the cooking process was the significant decrease (*p* < 0.05) in *n*-3 PUFA (overall EPA and DHA) and significant increase in *n*-6 PUFA, from the raw sample to the five cooking methods, when expressed in percentage and on a dry weight basis (*p* < 0.05) (Table 1 and Table 3, Figure 3). In particular, *n*-3 PUFA decreased after pan frying by 2.5 times. Similar results were shown by a number of studies, e.g., by Bejaoui [32], for *Ruditapes decussatus* after four cooking processes, by Felici [31], for *Crassostrea gigas*, by Kalogeropulos [23], for *M. galloprovincialis*, for fish and cephalopods and by Zhang [45], for grass carp (*Ctenopharynyodon idellus*). On the other hand, other studies showed that of the various cooking methods (boiling, frying, grilling, oven cooking, microwaving), only frying determined a significant decrease of *n*-3 fatty acids [29,46].

Owing to the higher moisture content, on a ww basis, a portion of 100 g of raw sample contained 356.48 mg of *n*-3, significantly lower than the other cooked mussels (*p* < 0.05) (Table 2).

Hence, the highest PUFA content observed in fried mussel was due to the highest *n*-6 PUFAs because of the significant uptake of sunflower oil rich in linoleic acid (C18:2 *n*-6) and concomitant loss of moisture by evaporation [35]. In particular, *n*-6 PUFAs of fried mussels accounted to 6769.18 mg/100 g dw (42.53% of total FAs), about 18-fold higher content of *n*-6 PUFAs of raw mussels, which showed a value of 374.70 mg/100 g dw (5.48% of total FAs) (Table 1 and Table 3). This result was in full agreement with other authors who investigated the effect of frying on seafood [23,24,25,32]. Therefore, oils rich in *n*-6 PUFA should be avoided in pan frying, because the quality of fried food depends also on the quality of the frying oil.

Another important *n*-6 fatty acid was arachidonic acid (ARA), which was shown, on a dry weight basis, to be affected by the cooking process, with the highest value in grilled mussels and the lowest in fried samples; no significant differences were found between raw and microwave cooking.

Marine organisms are known to be the main source of PUFAs, and humans can meet the need for essential fatty acids, particularly of the *n*-3 series EPA and DHA, mainly by consuming fish and seafood in general [47]. Marine phytoplankton and the small aquatic plant cells are the main producers of *n*-3 PUFAs and represent food for many aquatic organisms, including fish and shellfish, that are components of the human diet. Epidemiological studies have shown that *n*-3 fatty acid intake is evidently crucial for human health, being involved in the prevention of many diseases, and it may help in the amelioration of cardiovascular disorders [48]. Most health benefits of seafood are attributed to *n*-3 PUFA, EPA and DHA that display several properties such as antithrombotic, anti-inflammatory, antiarrhythmic and vasodilatory [48]. They are implied in the prevention of cognition decline, both age-associated and from cancer, in reducing mild hypertension, in lowering the incidence of diabetes [2], in photoreception (vision), in fetal neurodevelopment and cognitive development [49].

PUFAs, such as EPA and DHA, are known to be the most sensitive to oxidation during culinary treatments due to the higher degree of unsaturation that makes them the most unstable fatty acids [20]. Indeed, in this study, EPA and DHA significantly decreased during all cooking processes. EPA showed the highest content in raw sample, on a dry weight basis, accounting for 884.52 mg/100 g dw (12.93%), while the maximum loss was observed in fried mussels, showing a value of 388.49 mg/100 g dw (2.44%) (Table 1 and Table 3, Figure 3). The cooked mussels that showed a lower, although significant, reduction of EPA compared to the raw sample were boiled (772.23 mg/100 g dw) and grilled (768.18 mg/100 g dw)—no difference between them. The trend of DHA proportion was the same as EPA (Table 3), showing the highest content in raw sample, 760.81 mg/100 g dw (11.12%) and markedly decreased in fried sample, accounting for 344.41 mg/100 g dw (only accounted for 2.16%). Boiled sample, with a value of 700.64 mg/100 g dw (10.08%), did not differ significantly from the raw sample (*p* > 0.05). Previous studies, covering a variety of fish species, have reported significantly lower EPA and DHA content after frying [46]. Obviously, when expressed as wet weight, EPA and DHA showed the lowest values in raw mussels, for the high moisture content.

### 3.3. Lipid Nutritional Quality Indices

Cooking processes can cause a reduction of the nutritional value of seafood, mainly due to heat-induced oxidation of the favorable *n*-3 PUFAs and the addition of exogenous oil that can modify the original FA profile of the specimens [22].

Considering the results of *n*-3 and *n*-6 PUFAs, the *n*-3/*n*-6 ratio markedly decreased (*p* < 0.05) from raw (6.01) to cooked mussels (Table 4, Figure 3). In particular, the *n*-3/*n*-6 ratio was severely altered in the case of fried samples, which showed the lowest *n*-3/*n*-6 ratio (0.15), in agreement with many authors who state that the exchange of fatty acids between food and cooking oil causes a significant loss of some important FAs such as *n*-3 EPA and DHA [50]. Grilled, boiled, microwaved and oven-cooked samples showed slight variation among them (range 3.23–3.84), although significant (*p* < 0.05). These findings agree with other studies, for example, Ghribi [12] reported a decrease of *n*-3/*n*-6 ratio in *Arca noae* after cooking, from 2.66 in raw sample to 1.85 (boiled), 1.38 (grilled) and 0.27 (fried). Felici [31], for *Crassostrea gigas,* also reported a significant decrease from 5.33 in raw to 3.58 and 3.29 in samples cooked in olive oil with garlic and in gratin samples, respectively. Bejaoui [32], for *Ruditapes decussatus,* showed a significant *n*-3/*n*-6 decrease from 6.9 in raw to 2.4 and 0.37 in grilled and fried clams, respectively. Similarly, Kalogeropoulos [23] and Otles and Sengor [24] found a decrease in fried *M. galloprovincialis* compared to the raw ones. Several studies on fish reported a similar decrease after the cooking process [34,35,46]. Hosseini [34], for kutum roach (*Rutilus frisii*), found the *n*-3/*n*-6 ratio decreased significantly from raw (3.89) to baked (1.89), microwaved (2.03) and fried samples (0.43) (*p* < 0.05), while boiling (3.61) did not differ from raw.

The *n*-3/*n*-6 ratio represents an important lipid quality index that more than any other highlights the alteration of fatty acids during the different cooking methods. The dietary importance of the *n*-3/*n*-6 ratio has long been known and several studies report that an equilibrate ratio can help in the prevention and treatment of many diseases. According to Biandolino [5] and Prato [9], bivalve species are characterized by elevated *n*-3/*n*-6 ratio, thus contributing to raise the nutritional value of this product.

However, eating habits have dramatically changed over the years and today the Western diet is low in *n*-3 and excessive in *n*-6 PUFAs, resulting in an unhealthy *n*-6/*n*-3 ratio of 17:1 or 20:1 [51], instead of the recommended ratio of 4 to 1. This is due to a low consumption of fish and the application of commercial fodder containing high amounts of *n*-6 and low levels of *n*-3. Several studies suggest that this unbalanced ratio in favor of *n*-6 PUFAs is prothrombotic and proinflammatory, and the inflammation constitutes the basis of many chronic pathologies such as cardiovascular disease, obesity, diabetes, arthritis, atherosclerosis, cancer and autoimmune conditions [51]. Simopoulos [51] proposed the *n*-3/ *n*-6 ratio in the range of 0.25 to 1.0 as a dietary intake standard. Food and Agriculture Organization FAO/WHO [52] recommend an *n*-3/*n*-6 ratio in the range of 1:8 and 2:5. Although the cooking process decreased this ratio, the results from this study, for both raw and cooked samples, except for fried sample, are better than the above recommended standards, indicating that also when cooked, the mussels are good for consumption. As regards the *n*-6/*n*-3 PUFAs ratio that must not exceed 4, it can be observed that in this study, raw and all cooked mussels, except fried (6.88), exhibited values far below the recommended ratio (Table 2). The *n*-6/*n*-3 ratio can be improved by increasing the *n*-3 PUFA and not by decreasing the *n*-6 PUFA.

Some researchers consider the ARA/EPA ratio as a better nutritional quality index than the *n*-3/*n*-6 ratio. An increase of the ARA/EPA ratio reduces the nutritional value of the product [53]. All cooking methods investigated in this study had a significant effect on this index, from 0.25 in raw sample to the highest value in grilled sample (0.46) (*p* < 0.05) (Table 4).

Another approach to evaluate the dietary quality of the lipid fraction is the PUFA/SFA ratio, which significantly increased in fried sample (3.56) due to the high content of *n*-6 PUFA in sunflower oil that migrated in the mussel tissue (Table 4, Figure 3). The other cooking methods did not affect this ratio (*p* < 0.05), showing a similar value to that of raw sample (0.92). The recommended minimum value of PUFA/SFA ratio is 0.45 and a ratio lower than this value is not favorable for human health, according to the Department of Health and Social Security [54].

However, the PUFA/SFA index should be considered with caution, as it does not take into account the important metabolic effects of MUFAs and the fact that some SFAs such as stearic acid do not increase plasma cholesterol. Indeed, in this study, another index was used in which MUFA was added and stearic acid was excluded from the saturated fraction. MUFA + PUFA/SFA-C18:0 values were significantly increased in cooked samples compared to raw sample (*p* < 0.05); even in between the cooked mussels—boiled and microwaved—and raw, the difference was lower (Table 4).

The atherogenic and thrombogenic indices were proposed by Ulbricht and Southgate [27] to evaluate the potential of food to influence the incidence of coronary heart disease. In particular, they provide an indication on the nutritional quality of lipids, with low values of AI and TI showing healthier food, having better nutritional quality of fatty acids (antiatherogenic and antithrombogenic FAs) and, consequently, greater potential for preventing the onset of coronary diseases [20]. The raw *M. galloprovincialis* showed values of AI and TI of 1.09 and 0.34, respectively (Table 4). These findings agree with those obtained by Biandolino [5] on the same bivalve species with values of 1.0 (AI) and 0.34 (TI) and on other bivalves such as *Arca noae*, *Flexopecten glaber*, *Limaria tuberculata*, *Mimachlamys varia*, *Modiolus barbatus*, *Ostrea edulis* and *Solen marginatus*.

In the present study, cooking process affected AI, showing a significant decrease in cooked samples compared to raw ones, especially in fried sample which showed the lowest value (0.19) (Table 4). Since AI is the ratio between some SFA and MUFA + PUFA (*n*-3 and *n*-6), the frying oil resulted in a great decrease of AI value. On the other hand, the higher AI value in raw sample was due to the high SFA content (atherogenic myristic acid) and low *n*-6 PUFA. Kalogeropoulos [23], for *M. galloprovincialis* fried in olive oil, also reported a significant reduction of AI in fried sample (0.30) compared to the raw state (0.73). Ghribi [12], for Noah’s Ark, reported AI values that were significantly different between raw (1.18) and fried sample (olive oil) (0.42), but was stable with other types of cooking, 1.22 for steamed, 1.22 for boiled and 1.13 for grilled. Bejaoui [32], for *R. decussatus,* found a decrease of AI values from raw (0.46) to grilled ones (0.31), but an increase for fried sample (0.86).

As with AI, the thrombogenic index(TI) was significantly lowest in fried sample (0.23), followed by grilled ones (0.31). Boiled, microwaved and oven-cooked samples did not show differences compared to raw sample (0.34) (*p* > 0.05) (Table 4). Kalogeropoulos [23] did not show a significant difference of TI between raw sample (0.25) and fried sample (0.30). Ghribi [12] for *A. noae* reported higher TI values compared to *M. galloprovincialis* from this study; however, they observed an increase of TI from raw (0.42) to fried samples (0.54 TI).

Since in the determination of this index there was a higher incidence of *n*-3 PUFA in all samples of this study (raw and cooked) except fried; the higher *n*-3 fatty acid content, and consequently the higher *n*-3/*n*-6 fatty acid ratio, contributed to lower thrombogenic index. As AI and TI are indicators of promotion and protection against coronary heart diseases, the low values exhibited by the fried sample would suggest a high cardioprotective effect. These data must be considered with caution, although evidence regarding the association between consumption of fried foods and the risk of coronary artery disease (CAD) is limited and conflicting. Literature data have reported values of AI from 0.10 to 2.37 and TI from 0.01 to 1.18 for different seafoods, including bivalves [12,23,25].

As regards the hypocholesterolemic/hypercholesterolemic fatty acid ratio, lower values of H/H are considered deleterious to human health. This ratio considers specific effects that single fatty acids might have on cholesterol metabolism, and high H/H values are desirable for human benefit. The results showed that this ratio increases significantly from 1.04 in raw sample to grilled (1.39) and fried (6.33) samples and therefore raw is superior in terms of cardiovascular protection. H/H increases slightly in all remaining cooked mussels compared to raw ones, although not significantly (*p* > 0.05) (Table 4). Surprisingly, raw mussels had the least favorable H/H ratio while fried had the most favorable. The reasons were the significantly higher share of myristic acid in the fat of raw mussels and high C18:2 *n*-6 and 18.1 *n*-9 in fried sample (Table 3). Hosseini [34] found, for the fish *Rutilus frisii* (kutum), a H/H ratio more favorable in fried sample (4.83) than in raw ones (3.59).

All these lipid indices (Table 4) highlight that the consumption of the raw and cooked mussels promotes reduction of the risk of ischemic heart disease and arterial hypertension, platelet aggregation, and prevents some cardiovascular disorders. There is evidence that in the assessment of cardiovascular disease risk, the type of fat is more important than the total amount of fat.

#### EPA + DHA Intake

The EPA + DHA sum is one of the most important lipid nutritional quality indices. In this study, all cooking methods caused a significant decrease (*p* < 0.05) of EPA + DHA. The lowest value was observed in fried sample, when expressed on the dry weight basis, with 732.91 mg/100 g (4.60% of total FAs). A similar reduction of EPA and DHA was found by Candela [44] in mackerel and sardines after frying in sunflower oil. As above reported, there are a number of pathologies in which the *n*-3 PUFA, particularly EPA and DHA, play an important role, thus reflecting the importance of ensuring their adequate dietary intake. There are a series of nutritional guidelines developed by various health scientific agencies and national and international organizations that provide recommendations for the dietary intake of EPA + DHA.

The European Food Safety Authority [55] recommends an intake of 250 mg of EPA + DHA for adults with an increased intake of DHA by an additional 100–200 mg/day for women during pregnancy or lactation. The Scientific Societies of Nutrition, in France, recommend 500 mg/day of EPA + DHA, with an intake of 120 mg/day minimum of DHA [56]. In the Netherlands, the Health Council [57] suggests an EPA + DHA intake of 450 mg/day, as does the United Kingdom Scientific Advisory Committee on Nutrition (2004). The Superior Health Council of Belgium [58] indicates an amount of approximately 667 mg/day.

As regards international organizations, the World Health Organization (WHO) recommends the consumption of between 0.3 and 0.5 g/day, while the International Society for the Study of Fatty Acids and Lipids (ISSFAL) recommends 500 mg/day and the North Atlantic Treaty Organization (NATO) recommends 800 mg/day. For individuals with coronary heart disease, 1 g/day of EPA + DHA has been shown to reduce coronary heart disease mortality.

In order to provide useful information to consumers about the potential nutritive value of cooked mussels, it was decided to refer to wet weight to estimate the amount of each cooked mussel to be consumed by an individual to obtain the daily intake of the two essential PUFAs (250–500 mg/day), as recommended by WHO [52] (Table 5).

Raw sample had the lowest EPA + DHA content (referred to 100 g on wet weight basis), followed by microwaved ones, due to the high moisture content. Therefore, it has been estimated that the recommended daily intake of EPA + DHA can be satisfied by eating about 96–192 g/day and 74–148 mg/day of raw and microwaved mussels, respectively (Table 5).

Cooking significantly affected the EPA + DHA content and oven-cooked mussel appeared to be the most valuable food as the essential PUFA source, because it contained a total of about 463.16 mg of EPA+ DHA/100 g wet weight. Indeed, in order to obtain a health benefit, a portion of only 54-108 g is enough to meet the daily requirement. A similar content of EPA + DHA was exhibited by grilled, boiled and fried samples (range 406–443 mg/100 g ww) (*p* < 0.05) (Table 5).

## 4. Conclusions

Based on the results obtained, it can be concluded that all cooking methods examined significantly affected the lipid and fatty acids profile of mussel *M. galloprovincialis*.

Although all processes were healthier than frying, the boiling and microwaving were found to be valuable and beneficial as these methods resulted in lower levels of less favorable fatty acids, such as *n*-6, already very abundant in the Western diet. However, oven-cooked mussels appeared to be the most valuable food for good fatty acid composition and highest EPA + DHA content (ww basis). Therefore, it may take its place among the type of cooking a consumer would prefer.

Frying greatly affected the fatty acid profile, with a drastic decrease in the ratio of *n*-3/*n*-6 PUFAs; therefore, it should be a discouraged method for cooking mussels because it is not favorable to human health.

The present study provides practical and useful information to consumers regarding the cooking method that would best preserve the nutritional value of a food.

## Figures and Tables

**Table 1:** Fatty acids profile (mg/100 g dry weight basis) of raw and cooked mussel.

	Raw	Grilled	Boiled	Microwaved	Oven-Cooked	Fried
C14:0	564.20 ± 38.55 ^d^	406.64 ± 13.42 ^c^	412.52 ± 0.82 ^c^	329.99 ± 11.37 ^b^	384.91 ± 25.71 ^c^	217.16 ± 5.92 ^a^
C15:0	132.86 ± 8.32 ^c^	70.35 ± 12.78 ^a^	75.10 ± 4.23 ^a^	61.55 ± 3.17 ^a^	67.48 ± 4.19 ^a^	96.68 ± 15.93 ^b^
C16:0	1932.32 ± 96.58 ^c^	1571.61 ± 27.78 ^a^	1740.57 ± 46.83 ^b^	1464.31 ± 12.45 ^a^	1735.42 ± 109.19 ^b^	1740.13 ± 86.70 ^b^
C17:0	150.93 ± 5.73 ^b^	126.03 ± 45.30 ^ab^	136.24 ± 18.71 ^ab^	149.21 ± 2.38 ^b^	102.24 ± 13.23 ^a^	nd
C18:0	220.92 ± 21.94 ^b^	292.17 ± 3.41 ^d^	265.25 ± 7.89 ^c^	227.49 ± 8.13 ^b^	280.36 ± 4.21 ^cd^	157.98 ± 18.95 ^a^
C14:1	nd	nd	166.55 ± 9.13	nd	nd	nd
C15:1	nd	64.77 ± 4.76 ^b^	nd	41.34 ± 14.84 ^b^	11.15 ± 5.23 ^a^	60.65 ± 52.94 ^b^
C16:1	578.38 ± 31.51 ^b^	903.89 ± 25.76 ^d^	895.32 ± 7.65 ^d^	717.74 ± 8.27 ^c^	877.54 ± 59.64 ^d^	497.80 ± 30.56 ^a^
C17:1	85.53 ± 9.17 ^d^	73.69 ± 1.20 ^cd^	53.68 ± 7.09 ^b^	61.94 ± 5.60 ^bc^	78.49 ± 12.63 ^d^	37.29 ± 3.51 ^a^
C18:1*n*-7		240.08 ± 67.34 ^b^	144.74 ± 10.25 ^a^	123.25 ± 9.76 ^a^	353.24 ± 85.07 ^c^	579.85 ± 10.74 ^d^
C18:1*n*-9c	308.07 ± 18.70 ^a^	365.27 ± 1.22 ^a^	215.51 ± 2.43 ^a^	309.99 ± 10.84 ^a^	389.90 ± 17.98 ^a^	4596.80 ± 392.25 ^b^
C20:1*n*-9	117.99 ± 6.34 ^b^	144.58 ± 1.81 ^c^	163.90 ± 1.98 ^d^	122.76 ± 1.42 ^b^	159.36 ± 10.93 ^d^	79.97 ± 15.31 ^a^
C18:2*n*-6c	120.95 ± 5.50 ^a^	206.93 ± 10.51 ^b^	214.18 ± 4.79 ^b^	185.38 ± 6.08 ^b^	222.21 ± 19.88 ^b^	6615.22 ± 714.80 ^c^
C18:3*n*-6	nd	26.27 ± 4.00	nd	nd	nd	nd
C18:3*n*-3	211.25 ± 4.09 ^a^	316.37 ± 13.00 ^c^	337.70 ± 10.28 ^c^	398.49 ± 7.04 ^d^	473.97 ± 20.42 ^e^	238.99 ± 13.37 ^b^
C18:4*n*-3	263.69 ± 12.31 ^d^	139.12 ± 4.96 ^c^	124.00 ± 3.86 ^b^	nd	54.78 ± 9.08 ^a^	nd
C22:0 + 20:3*n*-6	31.38 ± 3.59 ^bc^	32.82 ± 2.76 ^c^	23.17 ± 3.92 ^a^	24.48 ± 1.61 ^ab^	36.61 ± 8.34^c^	nd
C20:3n-3 + 22:1	30.41 ± 4.08 ^b^	24.64 ± 1.22 ^ab^	18.68 ± 2.86 ^a^	nd	28.85 ± 9.91 ^b^	nd
C20:4*n*-6	222.37 ± 8.11 ^b^	351.50 ± 7.76 ^d^	286.07 ± 7.58 ^c^	233.73 ± 0.55 ^b^	294.28 ± 4.76 ^c^	153.96 ± 23.40 ^a^
C22:2	121.76 ± 14.03 ^bc^	210.26 ± 8.12 ^d^	143.04 ± 16.00 ^c^	114.08 ± 14.80 ^b^	130.14 ± 2.22 ^bc^	81.71 ± 22.60 ^a^
C20:5n-3 (EPA)	884.52 ± 50.45 ^e^	768.18 ± 29.56 ^d^	772.23 ± 24.80 ^d^	612.99 ± 31.41 ^b^	706.43 ± 15.50 ^c^	388.49 ± 31.71 ^a^
C22:5*n*-3	100.65 ± 5.25 ^d^	61.42 ± 4.07 ^c^	58.39 ± 2.43 ^bc^	53.17 ± 7.51 ^bc^	51.27 ± 3.19 ^b^	27.39 ± 5.08 ^a^
C22:6*n*-3 (DHA)	760.81 ± 42.27 ^d^	684.25 ± 36.11 ^c^	700.64 ± 39.14 ^cd^	550.10 ± 20.70 ^b^	579.55 ± 32.17 ^b^	344.41 ± 70.24 ^a^
∑SFA	3001.23 ± 66.67 ^d^	2466.80 ± 98.46 ^b^	2629.68 ± 42.99 ^c^	2232.54 ± 27.05 ^a^	2570.41 ± 117.79 ^bc^	2211.96 ± 104.23 ^a^
∑MUFA	1089.97 ± 29.86 ^a^	1792.29 ± 72.67 ^c^	1639.70 ± 25.17 ^bc^	1377.02 ± 25.81 ^ab^	1869.69 ± 51.90 ^c^	5852.36 ± 461.90 ^d^
∑ PUFA	2747.79 ± 47.60 ^b^	2821.76 ± 50.79 ^b^	2678.12 ± 67.83 ^b^	2172.45 ± 47.98 ^a^	2578.10 ± 78.83 ^ab^	7850.18 ± 557.55 ^c^
∑ *n*-3	2251.33 ± 72.32 ^d^	1993.99 ± 55.45 ^c^	2011.66 ± 64.43 ^c^	1614.75 ± 58.30 ^b^	1894.85 ± 75.87 ^c^	999.30 ± 117.26 ^a^
∑ *n*-6	374.70 ± 10.72 ^a^	617.52 ± 9.62 ^a^	523.42 ± 8.71 ^a^	443.60 ± 4.59 ^a^	553.11 ± 19.58 ^a^	6769.18 ± 691.40 ^b^
EPA + DHA	1645.33 ± 92.12 ^d^	1452.43 ± 65.53 ^c^	1472.87 ± 63.92 ^c^	1163.09 ± 50.69 ^b^	1285.98 ± 39.39 ^b^	732.91 ± 101.45 ^a^

All values are means of three separate replicates. Means with different letters (a, b, c, d, e) within each raw sample correspond to statistical differences (*p* < 0.05). SFA, saturated fatty acids; MUFA, monounsaturated fatty acids; PUFA, polyunsaturated fatty acids; nd, not detected; EPA, eicosapentaenoic acid; DHA, docosahexaenoic acid.

**Table 2:** Fatty acids profile (mg/100 g wet weight basis) of raw and cooked mussel. Fatty acids of sunflower oil used for frying (BF, before frying; AF, after frying).

	Raw	Grilled	Boiled	Microwaved	Oven-Cooked	Fried	Sunflower Oil BF	Sunflower Oil AF
C14:0	89.34 ± 6.10 ^a^	113.60 ± 3.75 ^b^	117.92 ± 0.24 ^b^	95.96 ± 3.31 ^a^	138.63 ± 9.26 ^c^	131.30 ± 3.58 ^c^	0.06	0.03
C16:0	305.97 ± 15.30 ^a^	439.07 ± 7.76 ^b^	497.53 ± 13.39 ^c^	425.82 ± 3.62 ^b^	625.03 ± 39.33 ^d^	1052.11 ± 52.42 ^e^	5.52	4.32
C18:0	34.98 ± 3.47 ^a^	81.62 ± 0.95 ^c^	75.82 ± 2.26 ^c^	66.15 ± 2.36 ^b^	100.97 ± 1.52 ^d^	95.52 ± 10.45 ^d^	2.81	1.97
C16:1	91.58 ± 4.97 ^a^	252.52 ± 7.20 ^c^	255.92 ± 2.19 ^c^	208.72 ± 2.40 ^b^	316.06 ± 21.48 ^d^	300.98 ± 18.48 ^d^	0.08	0.02
C18:1*n*-9	48.78 ± 2.96 ^a^	102.05 ± 0.34 ^a^	61.60 ± 0.69 ^a^	90.14 ± 3.15 ^a^	140.43 ± 6.48 ^a^	2779.31 ± 237.16 ^b^	30.21	13.64
C20:1*n*-9	18.68 ± 1.00 ^a^	40.39 ± 0.50 ^ab^	46.85 ± 0.57 ^ab^	35.70 ± 0.41 ^ab^	57.40 ± 3.94 ^c^	48.35 ± 9.26 ^b^	0.37	0.19
C18:2*n*-6c	19.15 ± 0.97 ^a^	57.81 ± 2.94 ^a^	61.22 ± 1.37 ^a^	53.91 ± 1.77 ^a^	80.03 ± 7.16 ^a^	3999.68 ± 432.18 ^b^	59.16	26.74
C18:3*n*-3	33.45 ± 0.65 ^a^	88.38 ± 3.63 ^b^	96.53 ± 2.94 ^b^	115.88 ± 2.05 ^c^	170.71 ± 7.36 ^e^	144.50 ± 8.08 ^d^	0.12	0.05
C20:4*n*-6	35.21 ± 1.28 ^a^	98.20 ± 2.17 ^de^	81.77 ± 2.17 ^c^	67.97 ± 0.16 ^b^	105.99 ± 1.72 ^e^	93.09 ± 13.15 ^d^		
C20:5*n*-3 (EPA)	140.06 ± 7.98 ^a^	214.61 ± 8.26 ^c^	220.74 ± 7.09 ^cd^	178.26 ± 9.14 ^b^	254.43 ± 5.58 ^e^	234.89 ± 19.18 ^d^	0.19	0.07
C22:5*n*-3	15.94 ± 0.83 ^a^	17.16 ± 1.14 ^a^	16.69 ± 0.69 ^a^	15.46 ± 2.19 ^a^	18.47 ± 1.15 ^a^	16.56 ± 3.07 ^a^		
C22:6*n*-3 (DHA)	120.47 ± 6.69 ^a^	191.16 ± 10.1 ^bc^	200.27 ± 11.19 ^c^	159.97 ± 6.02 ^b^	208.73 ± 11.59 ^c^	208.24 ± 42.47 ^c^		
∑SFA	475.22 ± 10.55 ^a^	689.16 ± 27.51 ^b^	751.68 ± 12.29 ^c^	649.22 ± 7.87 ^b^	925.77 ± 42.42 ^d^	1337.39 ± 63.02 ^e^	8.39	6.36
∑MUFA	172.59 ± 4.73 ^a^	500.72 ± 20.30 ^bc^	468.70 ± 7.19 ^b^	400.43 ± 7.51 ^b^	673.39 ± 18.69 ^c^	3538.44 ± 279.28 ^d^	30.66	13.85
∑ PUFA	435.09 ± 7.36 ^a^	788.32 ± 14.19 ^bc^	765.52 ± 19.39 ^bc^	631.74 ± 13.95 ^ab^	928.54 ± 28.39 ^c^	4746.37 ± 337.11 ^d^	59.47	26.86
∑ *n*-3	356.48 ± 11.45 ^a^	557.06 ± 15.49 ^c^	575.02 ± 18.42 ^c^	469.57 ± 16.95 ^b^	682.46 ± 27.33 ^d^	604.19 ± 70.90 ^c^	0.31	0.12
∑ *n*-6	59.33 ± 1.70 ^a^	172.52 ± 2.69 ^a^	149.62 ± 2.49 ^a^	129.00 ± 1.33 ^a^	199.21 ± 7.05 ^a^	4092.77 ± 418.03 ^b^	59.16	26.74
EPA + DHA	260.52 ± 14.58 ^a^	405.77 ± 18.31 ^c^	421.01 ± 18.27 ^cd^	338.23 ± 14.74 ^b^	463.16 ± 14.19 ^d^	443.13 ± 61.34 ^cd^		

All values are means of three separate replicates. Means with different letters (a, b, c, d, e) within each raw sample correspond to statistical differences (*p* < 0.05). SFA, saturated fatty acids; MUFA, monounsaturated fatty acids; PUFA, polyunsaturated fatty acids; nd, not detected; EPA, eicosapentaenoic acid; DHA, docosahexaenoic acid.

**Table 3:** Fatty acids profile (% of total FAs) of raw and cooked mussel.

	Raw	Grilled	Boiled	Microwaved	Oven-Cooked	Fried
C14:0	8.25 ± 0.56 ^c^	5.74 ± 0.19 ^b^	5.94 ± 0.01 ^b^	5.70 ± 0.20 ^b^	5.48 ± 0.36 ^b^	1.36 ± 0.04 ^a^
C15:0	1.94 ± 0.12 ^c^	0.99 ± 0.18 ^b^	1.08 ± 0.06 ^b^	1.06 ± 0.05 ^b^	0.96 ± 0.06 ^b^	0.61 ± 0.10 ^a^
C16:0	28.25 ± 1.41 ^d^	22.19 ± 0.39 ^b^	25.05 ± 0.67 ^c^	25.32 ± 0.21 ^c^	24.73 ± 1.55 ^c^	10.93 ± 0.54 ^a^
C17:0	2.20 ± 0.08 ^bc^	1.78 ± 0.64 ^ab^	1.96 ± 0.27 ^ab^	2.58 ± 0.04 ^c^	1.46 ± 0.19 ^a^	nd
C18:0	3.23 ± 0.32 ^b^	4.13 ± 0.05 ^d^	3.82 ± 0.11 ^c^	3.93 ± 0.14 ^cd^	3.99 ± 0.06 ^cd^	0.99 ± 0.11 ^a^
C14:1			2.40 ± 0.13			
C15:1		0.91 ± 0.07 ^c^		0.71 ± 0.26 ^bc^	0.16 ± 0.07 ^a^	0.57 ± 0.06 ^b^
C16:1	8.46 ± 0.46 ^b^	12.76 ± 0.36 ^c^	12.89 ± 0.11 ^c^	12.41 ± 0.14 ^c^	12.50 ± 0.85 ^c^	3.13 ± 0.19 ^a^
C17:1	1.25 ± 0.13 ^d^	1.04 ± 0.01 ^c^	0.77 ± 0.10 ^b^	1.07 ± 0.09 ^cd^	1.12 ± 0.18 ^cd^	0.23 ± 0.02 ^a^
C18:1*n*-7		3.39 ± 0.95 ^b^	2.08 ± 0.15 ^a^	2.13 ± 0.17 ^a^	5.03 ± 1.21 ^c^	3.64 ± 0.07 ^b^
C18:1*n*-9c	4.50 ± 0.27 ^ab^	5.16 ± 0.02 ^b^	3.10 ± 0.03 ^a^	5.36 ± 0.19 ^b^	5.55 ± 0.25 ^b^	28.88 ± 2.46 ^c^
C20:1*n*-9	1.72 ± 0.09 ^b^	2.04 ± 0.02 ^c^	2.36 ± 0.03 ^e^	2.12 ± 0.02 ^cd^	2.27 ± 0.15 ^de^	0.50 ± 0.09 ^a^
C18:2*n*-6c	1.77 ± 0.08 ^a^	2.92 ± 0.14 ^a^	3.08 ± 0.06 ^a^	3.21 ± 0.10 ^a^	3.17 ± 0.28 ^a^	41.57 ± 4.49 ^b^
C18:3*n*-6		0.37 ± 0.05				
C18:3*n*-3	3.09 ± 0.06 ^b^	4.47 ± 0.18 ^c^	4.86 ± 0.15 ^d^	6.89 ± 0.12 ^e^	6.75 ± 0.29 ^e^	1.50 ± 0.08 ^a^
C18:4*n*-3	3.85 ± 0.18 ^d^	1.96 ± 0.07 ^c^	1.78 ± 0.05 ^b^	0	0.78 ± 0.09 ^a^	0
C22:0 + 20:3*n*-6	0.46 ± 0.05 ^b^	0.46 ± 0.04 ^b^	0.33 ± 0.05 ^a^	0.42 ± 0.03 ^ab^	0.52 ± 0.11 ^b^	0
C20:3 n-3 + 22:1	0.44 ± 0.06 ^b^	0.35 ± 0.01 ^ab^	0.27 ± 0.04 ^a^	0	0.41 ± 0.14 ^b^	0
C20:4*n*-6	3.25 ± 0.11 ^b^	4.96 ± 0.10 ^d^	4.12 ± 0.11 ^c^	4.04 ± 0.01 ^c^	4.19 ± 0.06 ^c^	0.97 ± 0.14 ^a^
C22:2	1.78 ± 0.20 ^b^	2.97 ± 0.11 ^c^	2.06 ± 0.23 ^b^	1.97 ± 0.25 ^b^	1.85 ± 0.03 ^b^	0.51 ± 0.14 ^a^
C20:5*n*-3 (EPA)	12.93 ± 0.74 ^d^	10.85 ± 0.42 ^bc^	11.11 ± 0.35 ^c^	10.60 ± 0.54 ^bc^	10.06 ± 0.22 ^b^	2.44 ± 0.20 ^a^
C22:5*n*-3	1.47 ± 0.08 ^d^	0.87 ± 0.06 ^c^	0.84 ± 0.03 ^bc^	0.92 ± 0.13 ^c^	0.73 ± 0.04 ^b^	0.17 ± 0.03 ^a^
C22:6*n*-3 (DHA)	11.12 ± 0.62 ^d^	9.66 ± 0.51 ^c^	10.08 ± 0.56^c^	9.51 ± 0.35 ^c^	8.26 ± 0.45 ^b^	2.16 ± 0.44 ^a^
∑SAFA	43.88 ± 0.97 ^e^	34.84 ± 1.39 ^b^	37.85 ± 0.62 ^cd^	38.61 ± 0.47 ^d^	36.62 ± 1.68 ^bc^	13.90 ± 0.65 ^a^
∑MUFA	15.94 ± 0.44 ^a^	25.31 ± 1.02 ^bc^	23.60 ± 0.36 ^b^	23.81 ± 0.45 ^b^	26.64 ± 0.74 ^c^	36.77 ± 2.90 ^d^
∑ PUFA	40.18 ± 0.70 ^b^	39.85 ± 0.72 ^b^	38.55 ± 0.98 ^ab^	37.57 ± 0.83 ^ab^	36.73 ± 1.12 ^a^	49.33 ± 3.50 ^c^
∑ *n*-3	32.92 ± 1.06 ^d^	28.16 ± 0.78 ^bc^	28.95 ± 0.92 ^c^	27.93 ± 1.01 ^bc^	27.00 ± 1.08 ^b^	6.28 ± 0.74 ^a^
∑ *n*-6	5.48 ± 0.15 ^a^	8.72 ± 0.13 ^b^	7.53 ± 0.12 ^ab^	7.67 ± 0.07 ^ab^	7.88 ± 0.27 ^ab^	42.53 ± 4.34 ^c^
EPA + DHA	24.06 ± 1.34 ^d^	20.51 ± 0.92 ^c^	21.20 ± 0.92 ^c^	20.11 ± 0.87 ^c^	18.32 ± 0.56 ^b^	4.60 ± 0.63 ^a^

All values are means of three separate replicates. Means with different letters (a, b, c, d, e) within each raw sample correspond to statistical differences (*p* < 0.05). SFA, saturated fatty acids; MUFA, monounsaturated fatty acids; PUFA, polyunsaturated fatty acids; nd, not detected; EPA, eicosapentaenoic acid; DHA, docosahexaenoic acid.

**Table 4:** Nutritional quality indexes of raw and cooked mussels *M. galloprovincialis*. Values are means ± standard deviations.

	Raw	Grilled	Boiled	Microwaved	Oven-Cooked	Fryed
*n*-3/*n*-6	6.01 ± 0.37 ^e^	3.23 ± 0.13 ^b^	3.84 ± 0.10 ^d^	3.64 ± 0.14 ^cd^	3.43 ± 0.17 ^bc^	0.15 ± 0.03 ^a^
*n*-6/*n*-3	0.17 ± 0.00 ^a^	0.31 ± 0.01 ^c^	0.26 ± 0.01 ^b^	0.27 ± 0.01 ^ab^	0.29 ± 0.01 ^bc^	6.88 ± 1.40 ^d^
PUFA/SFA	0.92 ± 0.03 ^a^	1.15 ± 0.06 ^a^	1.02 ± 0.04 ^a^	0.97 ± 0.03 ^a^	1.00 ± 0.07 ^a^	3.56 ± 0.40 ^b^
EPA/DHA	1.16 ± 0.01 ^ab^	1.12 ± 0.02 ^ab^	1.10 ± 0.02 ^a^	1.11 ± 0.03 ^ab^	1.22 ± 0.07 ^b^	1.15 ± 0.14 ^ab^
ARA/DHA	0.29 ± 0.02 ^a^	0.51 ± 0.02 ^d^	0.41 ± 0.01 ^b^	0.42 ± 0.01 ^bc^	0.51 ± 0.02 ^d^	0.45 ± 0.03 ^c^
ARA/EPA	0.25 ± 0.02 ^a^	0.46 ± 0.01 ^d^	0.37 ± 0.00 ^b^	0.38 ± 0.02 ^b^	0.42 ± 0.01 ^c^	0.39 ± 0.02 ^bc^
(MUFA + PUFA)/SFA - C18:0	1.38 ± 0.06 ^a^	2.12 ± 0.14 ^c^	1.83 ± 0.04 ^b^	1.77 ± 0.03 ^b^	1.95 ± 0.15 ^bc^	6.68 ± 0.33 ^d^
AI	1.09 ± 0.03 ^d^	0.69 ± 0.03 ^b^	0.78 ± 0.02 ^c^	0.78 ± 0.02 ^c^	0.74 ± 0.06 ^bc^	0.19 ± 0.01 ^a^
TI	0.34 ± 0.01 ^c^	0.31 ± 0.01 ^b^	0.33 ± 0.01 ^bc^	0.34 ± 0.01 ^c^	0.34 ± 0.03 ^bc^	0.23 ± 0.01 ^a^
HH	1.04 ± 0.06 ^a^	1.39 ± 0.05 ^b^	1.20 ± 0.05 ^ab^	1.31 ± 0.04 ^ab^	1.29 ± 0.10 ^ab^	6.33 ± 0.37 ^c^

Means within the same row without a common lowercase letter differ significantly (*p* < 0.05). SFA, saturated fatty acids; MUFA, monounsaturated fatty acids; PUFA, polyunsaturated fatty acids; UNS, unsaturated fatty acids; EPA, eicosapentaenoic acid; DHA, docosahexaenoic acid; ARA, arachidonic acid; AI, Atherogenic Index; TI, Thrombogenicity Index; HH hypocholesterolaemic/hypercholesterolaemic fatty acid ratio.

**Table 5:** EPA + DHA (mg/100 g wet weight (ww) basis) content in *M. galloprovincialis,* raw and cooked; mussel portion needs to meet recommended daily intake of EPA + DHA (250–500 mg/day).

	EPA + DHA mg/100 g ww	Mussel Portion (g) 250 mg–500 mg/d
Raw	260.52 ± 14.58 ^a^	96.15 – 192.31 ^d^
Grilled	405.77 ± 18.31 ^c^	61.70–123.40 ^b^
Boiled	421.01 ± 18.27 ^cd^	59.45–118.90 ^ab^
Microwaved	338.23 ± 14.74 ^b^	74.00–148.00 ^c^
Oven-cooked	463.16 ± 14.19 ^d^	54.01–108.02 ^a^
Fried	443.13 ± 61.34 ^cd^	57.10–114.20 ^ab^

EPA eicosapentaenoic acid; DHA docosahexaenoic acid. Different letters (a, b, c, d) within the column denote significant differences among experimental groups.
